# Pan-cancer analysis of clinical significance and associated molecular features of glycolysis

**DOI:** 10.1080/21655979.2021.1955510

**Published:** 2021-07-24

**Authors:** Yi-chen Liu, Peng Lin, Yu-jia Zhao, Lin-Yong Wu, Yu-quan Wu, Jin-bo Peng, Yun He, Hong Yang

**Affiliations:** Department of Medical Ultrasound, The First Affiliated Hospital of Guangxi Medical University, Nanning, China

**Keywords:** Glycolysis, pan-cancer, multi-omics, immune evasion

## Abstract

Tumor glycolysis is a major promoter of carcinogenesis and cancer progression. Given its complex mechanisms and interactions, comprehensive analysis is needed to reveal its clinical significance and molecular features. On the basis of a well-established glycolysis gene expression signature, we quantified 8633 patients with different cancer types from the Cancer Genome Atlas (TCGA) and evaluated their prognostic associations. High tumor glycolytic activity correlated with inferior overall survival in the pan-cancer patients (hazard ratio: 1.70, 95% confidence interval: 1.20–2.40, P = 0.003). The prognostic value of glycolysis correlated with the molecular subtypes and was stable regardless of clinical parameters. The prognostic significance of glycolysis was validated using three independent datasets. In addition, genome, transcriptome, and proteome profiles were utilized to characterize the distinctive molecular features associated with glycolysis. Mechanistically, glycolysis fulfilled the fundamental needs of tumor proliferation in multiple ways. Exploration of the relationships between glycolysis and tumor-infiltrating immune cells showed that glycolysis enabled the immune evasion of tumor cells. Mammalian target of rapamycin (mTOR) inhibitors and dopamine receptor antagonists can effectively reverse the glycolytic status of cancers. Overall, our study provides an in-depth molecular understanding of tumor glycolysis and may have practical implications for clinical cancer therapy.

## GRAPHICAL ABSTRACT

**Figure:**
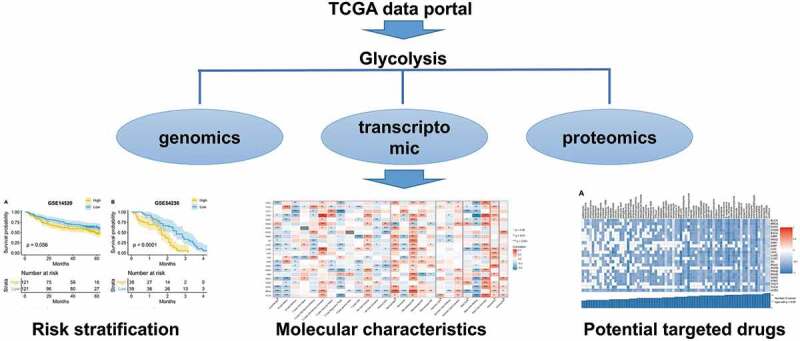

## Introduction

Carcinogenesis and tumor cell development are dependent on metabolic reprogramming to meet their energy and macronutrient requirements [1,2]. In alterations of metabolic reprogramming, the Warburg effect refers to the tendency of cancer cells to utilize glucose via glycolysis irrespective of oxygen availability [3,4]. Tumor glycolysis is a biological phenotype of most tumors and has served as a basis for cancer detection with positron emission tomography. Glycolysis is associated with advanced tumor progression, treatment resistance, and poor clinical outcomes [5,6]. It is also significantly linked to many cancer molecular characteristics, including proliferation, angiogenesis, and immune evasion [7,8]. Hence, targeting tumor glycolysis is important for cancer therapy [9]. However, a systematic characterization of the clinical and molecular characteristics of glycolysis is still needed.

In recent years, the multi-omics data from the Cancer Genome Atlas (TCGA) have driven the understanding of the molecular landscape of primary tumor beyond individual molecular platforms by integrating genomic, transcriptomic, and proteomic characteristics and clinicopathological parameters [10]. The tumor glycolysis status is also related to multiple layers of molecular alterations [11–14]. Many studies on the mechanisms of glycolysis have presented a clear picture of tumor characteristics. For example, upregulated tumor glycolysis was found to modulate T cell–mediated antitumor activity, thereby inhibiting melanoma patients’ response to adoptive T cell therapy [15]. Glycolysis can also enable the maintenance of strong tumorigenic activity on transcription factors, such as YAP/TAZ [16]. Furthermore, glycolytic metabolism modulates the translation of hypoxia-inducible factor 1-alpha (HIF1A) to control T cell responses to hypoxia [17]. Together, these studies establish that glycolysis can lead to multiple layers of molecular changes and thus plays a pivotal role in cancer development. However, the mechanistic details underlying the cancer glycolysis phenotype remain unclear.

Using multi-omics data, we quantify glycolysis and reveal its prognostic value from pan-cancer perspectives. By integrating multi-omics data with genome, transcriptome, and proteome data, we specify molecular characteristics related to glycolysis. Finally, we identify specific molecular compounds that may eventually result in novel therapy strategies that can reverse the glycolytic status of cancers. These approaches provide an unprecedented opportunity to explore the biological characteristics of tumors in great depth.

Our study aims to prove that glycolysis affects cancer progression by regulating the cell cycle, energy material synthesis, and cell proliferation in cancer, thereby providing an in-depth molecular understanding of tumor glycolysis and providing theoretical and technical support for clinical cancer treatment.

## Materials and methods

### Classification of tumor glycolysis scores across various cancer types

Molecular profiles, including oncogenic signaling pathways, messenger RNA (mRNA) expression, and protein expression profiles, were acquired from the TCGA pan-cancer project (https://gdc.cancer.gov/about-data/publications/pancanatlas). Genome-wide RNA sequencing (RNAseq) data profiles were obtained from the TCGA dataset using an Illumina HiSeq 2000 RNAseq system. Cancer-relevant proteins and phosphoproteins were detected by reverse-phase protein arrays. The gene signature for glycolysis (REACTOME_GLYCOLYSIS) was defined on the basis of 72 genes involved in glycolysis from the Molecular Signatures Database (MSigDB, http://software.broadinstitute.org/gsea/msigdb). Only primary tumor samples with an overall survival (OS) of no less than 30 days were used to obtain reliable results for survival analysis. Finally, we analyzed 23 TCGA nonhematologic cancer types with a sample size ≥ 100. The glycolysis score for each tumor sample across the cancer types was calculated using gene set variation analysis (GSVA) [18].

## Estimation of prognostic value of pan-cancer glycolysis

Univariate Cox analysis was performed to explore the relationship between the glycolysis scores and tumor patients’ OS. Then, meta-analysis was conducted using Stata 14.0 (Stata Corporation, College Station, TX, USA) to integrate the univariate Cox survival analysis results into each type of cancer. The heterogeneity between individual studies was evaluated by the Q test: I^2^ > 50% and/or P ≤ 0.05 indicated significant heterogeneity. The use of a random-effects or fixed-effects model was determined on the basis of the heterogeneity analysis results. Subgroup analyses stratified by age, gender, and tumor stage were performed to further explore variations in the effect of glycolysis on OS.

We performed consensus cluster analysis to identify a global pattern of glycolysis across the 23 types of cancers using the ConsensusClusterPlus package in the software R [19]. The patients were grouped into two clusters according to glycolysis-related genes using two-class K-means clustering with the Euclidean distance. Principal component analysis (PCA) was used to observe the patients in the two clusters. The OS difference between the clusters was calculated by the Kaplan-Meier (K-M) method and the log-rank test.

## Clinical and molecular characterizations related to glycolysis

We evaluated the glycolysis status for ten canonical signaling pathways with frequent genetic alterations: (1) cell cycle, (2) Hippo, (3) Myc, (4) Notch, (5) oxidative stress response/nuclear factor erythroid 2–related factor 2 (Nrf2), (6) phosphoinositide 3-kinase (PI3K), (7) receptor tyrosine kinase (RTK)/RAS/mitogen-activated protein (MAP) kinase, (8) transforming growth factor beta (TGF-β), (9) p53, and (10) β-catenin/Wnt signaling [20]. A tumor sample was found to have changed on the basis of genes involved in pathways that had recurrently altered and mutated positions, known functional gene fusions/rearrangements, and epigenetic silencing calls. Then, the Mann-Whitney U test was performed to estimate the difference in the glycolysis scores of the pathways with and without alterations.

We also computed the correlation between the glycolysis scores, mRNA, and protein expression profiles. Spearman’s rank correlation was used to assess the correlation between the glycolysis scores and molecular factors. The glycolysis-associated genes and proteins in each cancer type were determined as follows: Spearman’s correlation |coefficient| > 0.3 and P < 0.05. We found that the glycolysis-associated genes were significantly positively correlated with the glycolysis scores in at least 15 cancer types. These genes were further subjected to gene ontology (GO) classification analysis using the R package clusterProfiler [21]. To ascertain the functional interactions between these glycolysis-related genes and the identified hub genes, protein-protein interaction (PPI) networks were constructed using the Search Tool for the Retrieval of Interacting Genes/Proteins (STRING) database version 11.0 (required interaction score: > 0.9) [22].

## Exploration of relation between increased glycolysis and immune evasion

Growing evidence suggests that increased glycolysis actively interferes with immune cell functions. Thus, we examined the association between glycolysis and immune status. The tumor immune microenvironment is an executor of immunotherapy. Using the tool CIBERSORT, we obtained 22 types of immune cells in six classes from the TCGA pan-cancer project: (1) lymphocytes (naïve B cells, memory B cells, naïve CD4 T cells, resting memory CD4 T cells, activated memory CD4 T cells, T follicular helper cells, regulatory T cells [Tregs], gamma delta T cells, CD8 T cells, resting NK cells, activated NK cells, plasma cells); (2) macrophages (monocytes, M0 macrophages, M1 macrophages, M2 macrophages); (3) dendritic cells (resting dendritic cells, activated dendritic cells); (4) mast cells (resting mast cells, activated mast cells); (5) neutrophils; and (6) eosinophils [23].

Antigen-specific T cell receptor and B cell receptor repertoires are critical for the recognition of malignant cells and may reflect a robust antitumor response involving a large number of antigen-specific adaptive immune cells.

## Compounds targeting cancer glycolysis

To determine which molecules could be effective in cancer-inhibitory glycolytic activity, we performed connectivity map (Cmap) analysis [24]. Cmap is a public online database similar to the Gene Set Enrichment Analysis (GSEA) database, which predicts target drugs on the basis of a query signature. Gene symbols were mapped to the HG-U133A probe set GPL96 platform ID. We identified 500 genes most positively associated and 500 genes most negatively associated with the glycolysis scores to query drugs matching the ‘reference’ signature. Compounds with an enrichment score < 0 and a P-value < 0.05 were identified as glycolysis antagonist drugs.

## Results

The topic of the study which is the clinical significance of the carcinoma in glycolysis and related molecular characteristics analysis was to verify the role of glycolysis in cancer (regulating the cell cycle, energy material synthesis, cell proliferation, and cancer progression), thereby facilitating an in-depth molecular understanding of tumor glycolysis and providing theoretical and technical support for clinical cancer treatment. We quantified and evaluated prognostic associations in 8633 patients with different types of cancer in TCGA and validated the prognostic significance of glycolysis in three independent datasets.

## Increased tumor glycolytic activity as indicator of inferior survival in various cancers

**Figure 1.**
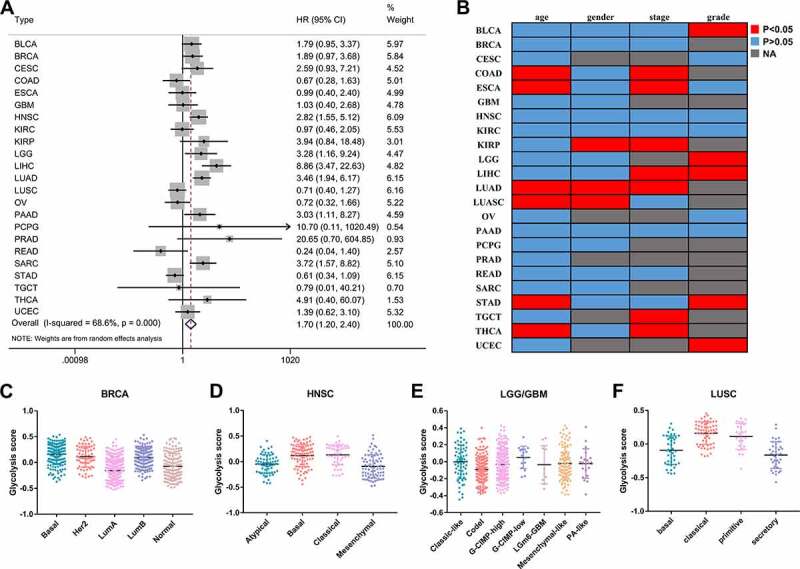
The prognostic value of glycolysis in pan-cancer patients

A total of 23 types of cancer in 8633 cases were identified in a survival analysis of glycolysis scores and OS. A 72-gene expression signature was used to calculate the glycolysis scores. The univariate Cox analysis showed that increased tumor glycolytic activity was significantly associated with a lower OS in six types of cancers: liver hepatocellular carcinoma (LIHC), lung adenocarcinoma (LUAD), head and neck squamous cell carcinoma (HNSC), sarcoma (SARC), brain low-grade glioma (LGG), and pancreatic adenocarcinoma (PAAD) (Table 1). A list of TCGA cancer type abbreviations is available at https://gdc.cancer.gov/resources-tcga-users/tcga-code-tables/tcga-study-abbreviations. To evaluate the prognostic value of glycolysis in the pan-cancer patients, we combined the hazard ratios (HRs) for the OS. Increased tumor glycolytic activity correlated with an inferior OS (HR: 1.70, 95% confidence interval: 1.20–2.40, P = 0.003; Figure 1a). Furthermore, we conducted subgroup analysis to observe the prognostic value of glycolysis in different clinicopathological parameters. We also analyzed the effects of glycolysis on OS by subgroups defined by age (< 60, ≥ 60 years), gender (female, male), and pathological stage (early stage, advanced stage). The subgroup analysis revealed that glycolysis is widespread and acts as an unfavorable factor for tumor patients’ prognosis (Table 2).

**Table 1.** Univariate cox analysis of glycolysis score

Study	N	Hazard ratio	95% confidence interval	P-value
BLCA	399	1.793	0.954–3.367	0.070
BRCA	1052	1.894	0.974–3.684	0.060
CESC	273	2.591	0.931–7.214	0.068
COAD	426	0.672	0.277–1.631	0.380
ESCA	178	0.985	0.404–2.399	0.973
GBM	146	1.034	0.400–2.676	0.945
HNSC	512	2.819	1.553–5.117	0.001
KIRC	518	0.972	0.460–2.053	0.940
KIRP	278	3.941	0.840–18.483	0.082
LGG	481	3.276	1.161–9.243	0.025
LIHC	343	8.862	3.471–22.628	5.00E-06
LUAD	492	3.460	1.940–6.173	2.60E-05
LUSC	474	0.714	0.401–1.271	0.252
OV	295	0.724	0.316–1.658	0.444
PAAD	172	3.034	1.113–8.271	0.030
PCPG	172	10.703	0.112–1020.485	0.308
PRAD	494	20.645	0.705–604.846	0.079
READ	153	0.243	0.042–1.403	0.114
SARC	255	3.723	1.571–8.825	0.003
STAD	375	0.609	0.341–1.087	0.093
TGCT	130	0.787	0.015–40.206	0.905
THCA	502	4.915	0.402–60.072	0.213
THYM	118	0.112	0.008–1.580	0.105
UCEC	513	1.389	0.622–3.100	0.423

**Table 2.** Subgroup analysis for the prognostic value of glycolysis score

Parameters	Types of cancer	No. of patients	HR (95%CI)	P-value	Model
Age					
Age <60	23	4034	1.82 (1.08–3.08)	0.025	Random effects
Age ≥60	22	4575	1.50 (1.01–2.22)	0.044	Random effects
Gender					
Female	21	4477	1.62 (1.25–2.11)	<0.001	Fixed effects
Male	20	4156	1.81 (1.11–2.93)	0.016	Random effects
Stage					
I/II	15	3631	1.82 (1.07–3.08)	0.027	Random effects
III/IV	14	2078	1.56 (1.17–2.10)	0.003	Fixed effects

(A) Forest plot of the correlation between glycolysis levels and OS of pan-cancer patients; (B) Heatmap shows P-values (including age, gender, pathologic stage, and histological grade) with significance (P < 0.05, red); (C–F) Highlight of tumor molecular subtypes that exhibit strong correlations with glycolysis.

To identify the clinical parameters associated with the glycolysis status in cancer, we compared the glycolysis scores between the different parameters. In many types of cancers, the glycolysis scores displayed a different status for each clinical feature (Figure 1b). For example, there were significant differences between the early and advanced stages in seven types of cancer. We further explored the status of glycolysis in different molecular subtypes of tumors (Figure 1c–F). In the case of breast cancer, the glycolysis score was the lowest in the luminal A subtype, which has the best prognosis.

**Figure 2.**
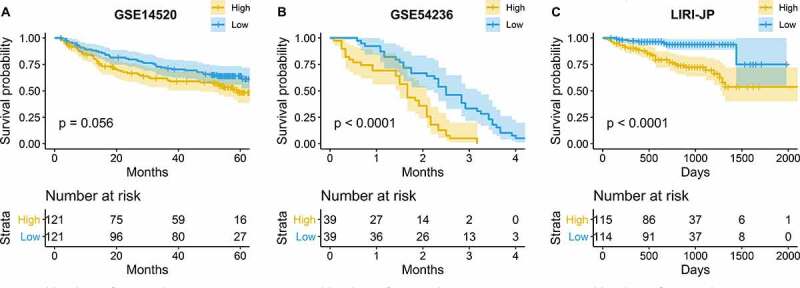
Validation of the prognostic value of hepatocellular carcinoma (HCC) in three independent cohorts

The prognostic value of glycolysis was the most significant in LIHC. Hence, three independent cohorts were used to validate the results: GSE14520 (N = 242) [25] and GSE54236 (N = 78) [26], which were acquired from the Gene Expression Omnibus (GEO) repository, and the LIRI-JP dataset (N = 229) [27], which was downloaded from the International Cancer Genome Consortium (ICGC) database. The glycolysis score was calculated using GSVA in hepatocellular carcinoma patients with an OS of no less than 30 days. On the basis of the median value of the glycolysis score, LIHC patients were divided into high- and low-glycolysis groups. The patients in the high-glycolysis group exhibited a lower OS (Figure 2).

(A) GSE14520; (B) GSE54236; (C) LIRI-JP.

## Global patterns of glycolysis signatures across cancer types

**Figure 3.**
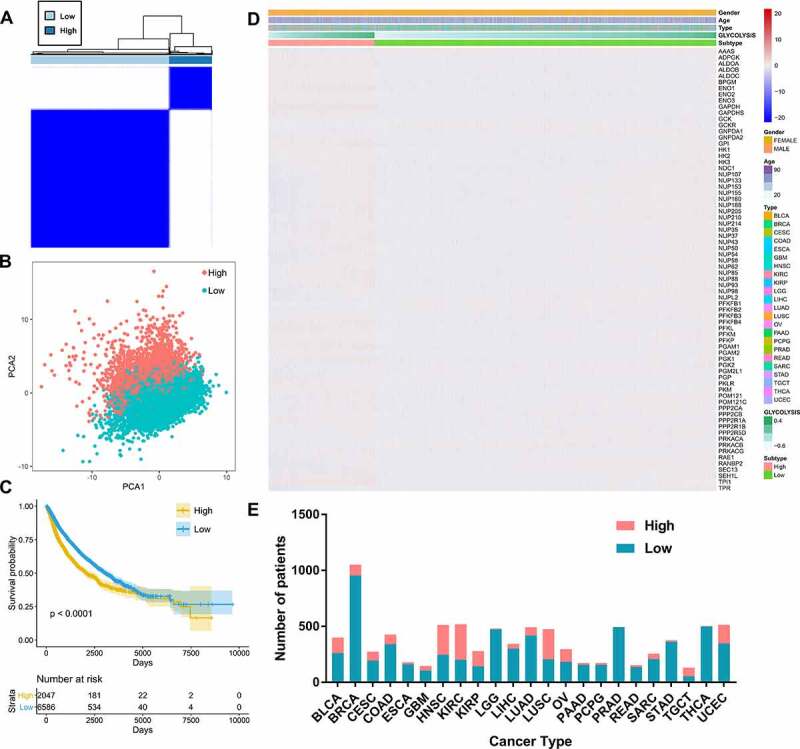
Exploration of metabolism-driven cancer types

According to the tumor expression levels of 72 glycolysis genes, 2047 (6585) patients were assigned to the group with high (low) expression of glycolysis-associated genes (Figure 3a). The PCA plot shows that the two clusters were markedly different (Figure 3b). As seen in the K-M survival plot, the patients in the high-glycolysis group had a poorer OS than that of the patients grouped into the low-glycolysis group (Figure 3c). The global expression profiles also indicated variations between the two groups; varied glycolysis levels significantly correlated with the different clinical parameters (Figure 3d). Notably, various types of tumors accounted for different ratios of the high-glycolysis group to the low-glycolysis group. This ratio was the highest in kidney renal clear cell carcinoma (KIRC) patients and lowest in prostate adenocarcinoma (PRAD) and thyroid cancer (THCA) patients (Figure 3e).

(A) Patients were divided into high- and low- glycolysis groups based on the gene expression of 72 signature genes generated by K-means clustering; (B) Principal component analysis plot indicates that the two subgroups have distinct glycolysis gene expression profiles; (C) Kaplan–Meier survival analysis curves for the two patient clusters; (D) Heatmap of the 8633 patients grouped by cluster, with annotations associated with each cluster; (E) The ratio of patients with high and low glycolysis in different cancer types.

## Glycolysis effects on multidimensional molecular factors

**Figure 4.**
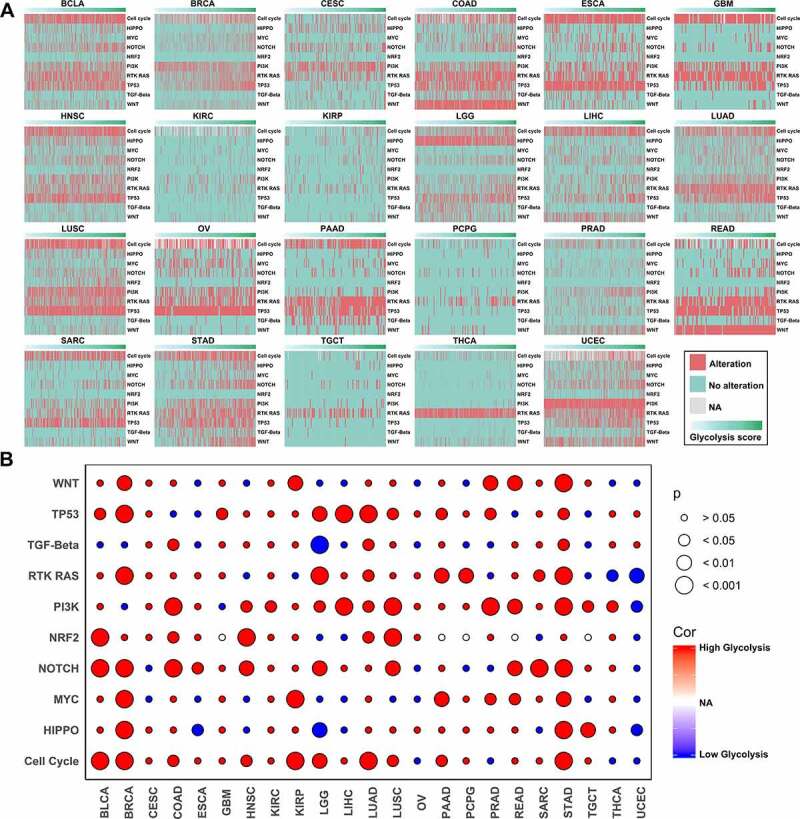
Associations between glycolysis and 10 oncogenic signaling pathway alterations

Signaling pathways are characterized by frequent somatic alterations and complex interactions with each other. Systematic characterization and exploration of the relationships between oncogenic signaling pathway alterations and glycolysis are therefore meaningful. In this study, the glycolysis scores were significantly different between many signaling pathways with and without alterations in different types of cancers (Figure 4a). Glycolysis was upregulated in the PI3K signaling pathway alterations group compared with the no-alterations group in 12 types of cancer; it was also upregulated in the cell cycle, tumor protein 53 (TP53), and Hippo signaling pathway alterations group compared with the no-alterations group in ten types of cancer (Figure 4b).

(A) Heatmap of glycolysis scores and pathway alterations; (B) Heatmap showing P-values (Mann–Whitney U test). Red indicates that the glycolysis score is upregulated, while blue indicates that it is downregulated in the pathway alteration group.

**Figure 5.**
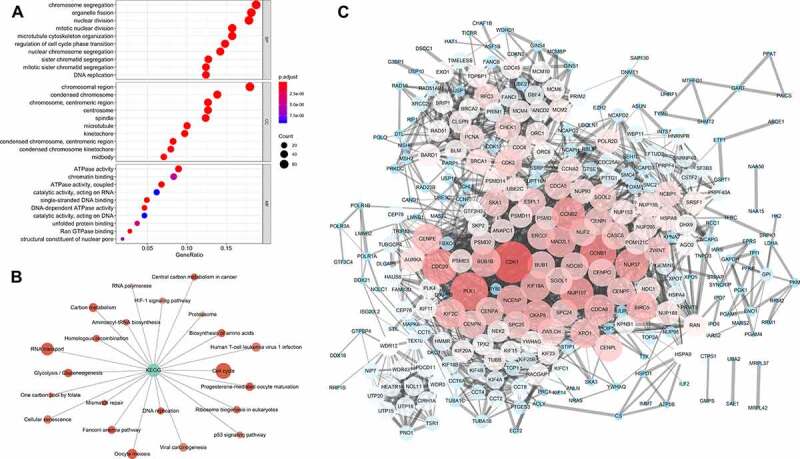
Gene functional enrichment analysis of glycolysis-associated genes

We also explored the glycolysis-associated mRNA expression profiles, as mRNA expression serves as a link between genetic alterations and protein actions. The genes that positively correlated with glycolysis in more than 15 cancer types were subjected to functional enrichment analysis. We found that these genes were mainly involved in cell proliferation–associated biological processes and energy-related molecular functions (Figure 5a). The pathway annotations indicated that ‘cell cycle’ was the most significant term related to the glycolysis alterations (Figure 5b). The PPI network analysis also suggested that some genes important for the cell cycle, such as CDK1 and PLK1, were cores in the network (Figure 5c).

(A) Gene ontology of glycolysis-associated genes; (B) Kyoto Encyclopedia of Genes and Genomes (KEGG) analysis of glycolysis-associated genes; (C) Protein–protein interaction (PPI) network of glycolysis-associated genes.

**Figure 6.**
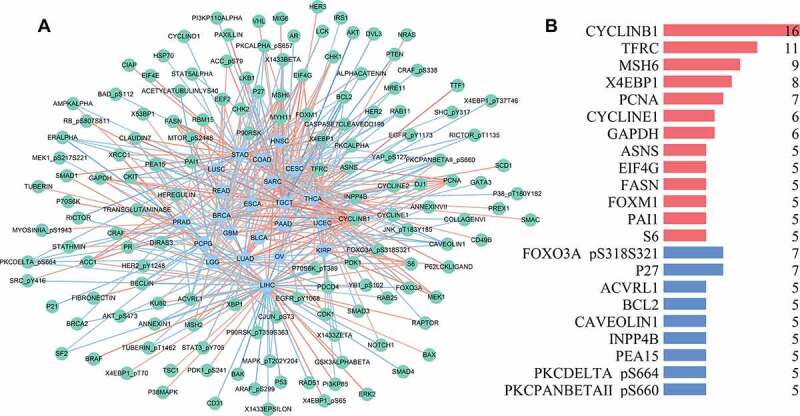
Correlation network of glycolysis-related proteins

At the protein level, some findings verified the results based on transcriptomic data and led to further discoveries (Figure 6a). Cyclin B1 was significantly positively associated with the glycolysis score in 16 types of tumors. FOXO3A PS318S321 and P27 were markedly negatively related to the glycolysis score in seven types of tumors (Figure 6b).

(A) Red lines show proteins positively related to glycolysis, while blue lines show proteins negatively related to glycolysis; (B) Bar plot indicates that cyclin B1 positively correlates with glycolysis in 16 types of cancer.

We also developed a network to show the relationships between glycolysis and proteins. On the basis of Spearman’s correlation analysis, the glycolysis-related proteins in each tumor type were identified and used to build the correlative network (Figure 6).

## Possible positive correlation of glycolytic activity with immune evasion

**Figure 7.**
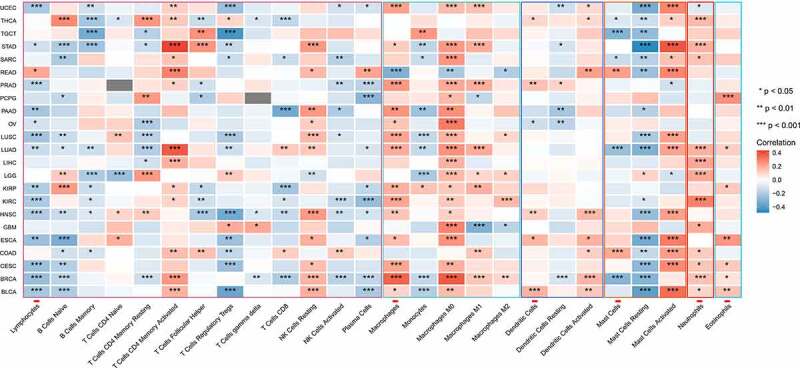
Associations between glycolysis and immune cell infiltrations

Tumor infiltration of lymphocytes is one of the key mechanisms in cancer progression and therapeutic response. Spearman’s correlation analysis showed that the glycolysis scores significantly negatively correlated with the infiltration of lymphocytes and positively correlated with the infiltration of macrophages and neutrophils (Figure 6). Programmed death-ligand 1 (PD-L1) expression was higher in the patients with high glycolysis scores than in those with low scores. Increased glycolysis generally correlated with increased T helper (TH) 2 cell infiltration, whereas TH1 cell infiltration showed the opposite correlation in most types of cancers (Figure 7).

## Compounds potentially capable of targeting glycolysis

**Figure 8.**
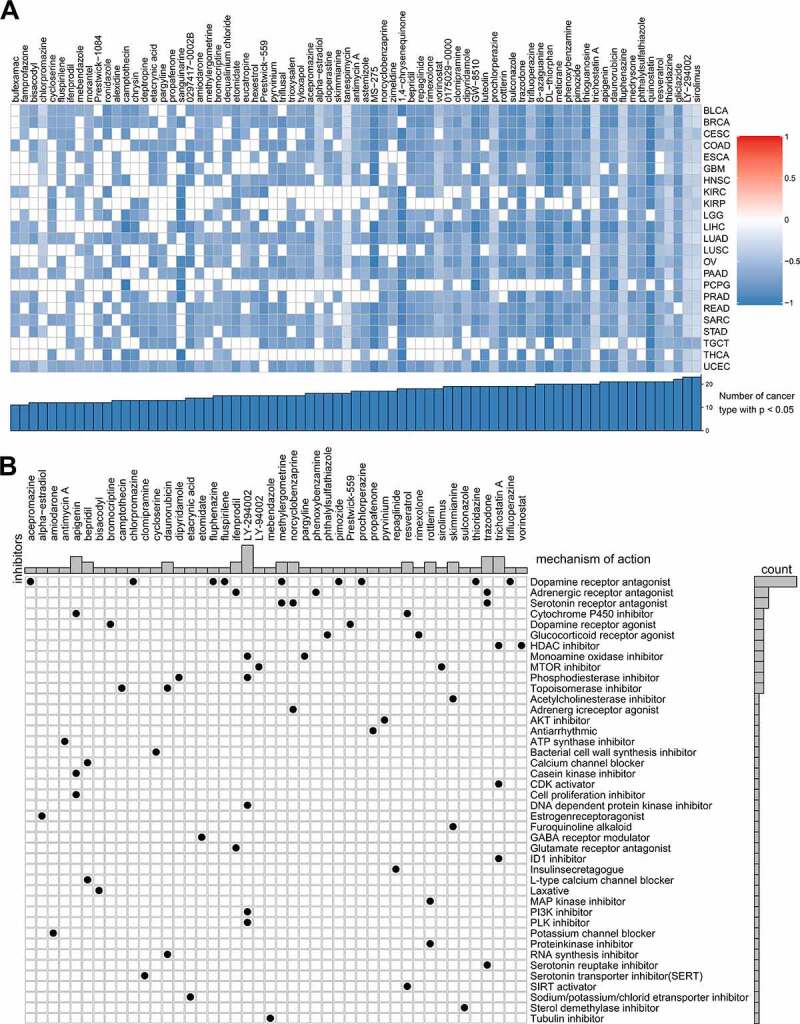
Correlation of glycolysis with drug resistance: connectivity map analysis

The online tool Cmap was used to discover the relationships between the genes, compounds, and biological processes and search for valuable targeted molecular compounds for glycolysis. We identified 75 that were closely related to glycolysis in not less than ten cancer types. Sirolimus and LY-294,002, as mTOR inhibitors, were found to target glycolysis in all 23 cancer types (Figure 8a). Cmap mode-of-action (MoA) analysis revealed that 42 mechanisms of action were shared by 44 of the compounds (Figure 8b). Nine compounds shared the MoA of dopamine receptor antagonists.

(A) Heatmap showing a negative enrichment score of each compound from Cmap for each cancer type; (B) Heatmap showing each Cmap compound (perturbagen) that shares mechanisms of action (rows) and sorted by descending number of compounds with shared mechanisms of action.

## Discussion

In this study, we systematically analyzed the prognostic value and effects of glycolysis on molecular signatures in 8633 primary human tumors across 23 cancer types. We gained insights into the relationship between glycolysis and low pan-cancer tumor OS. Our study provides a comprehensive view of glycolysis-associated molecular signatures, including genome, transcriptome, and proteome data. This work describes the relation between glycolysis and immune evasion from immune cell infiltration and immune-related indicators. Our exploration leads to potentially actionable compounds as candidates for alternative metabolism therapy for solid tumors. Our integrative analysis further suggests that glycolysis can impact tumors in many ways.

Metabolic reprogramming fulfills tumors’ energy/nutrient requirements and is considered a hallmark of cancer [2]. Previous studies state that a higher glycolysis level significantly correlates with a higher risk of adverse events or death in many types of cancers, including head and neck [28], lung [29], and esophageal [30]. With advancements in next-generation technologies and public cancer databases, it is now possible to comprehensively analyze the prognostic value of glycolysis in various cancer types. We found that increased tumor glycolytic activity is associated with poor clinical outcomes in the pan-cancer sample. This finding is consistent with recent work demonstrating that high glycolytic activity positively correlates with a higher death risk. The prognostic significance was stable in patients with different clinicopathological parameters. These results indicate fundamental and universal traits of glycolysis in cancer prognosis.

By integrating multi-omics data, we observed that oncogenic glycolysis is related to some molecular characteristics. Multi-omics analysis can link and integrate multilayered information and provide more reliable and accurate results than those of single-platform analysis. The first characteristic is perturbations in oncogenic signaling pathways. Several important signaling pathways are frequently genetically altered in cancer [31]. In the present study, patients with PI3K, cell cycle, and TP53 pathway alterations had higher glycolysis scores in multiple tumor types. At the transcriptome level, we identified glycolysis-associated genes, and gene functional enrichment analysis indicated essential cell cycle and proliferation processes. Proteomics analysis revealed that cell cycle regulatory protein cyclin B1 is an important participant in the glycolysis process. Different multi-omics data suggest that glycolysis is inextricably related to tumor proliferation. The most basic characteristic of cancer cells is continuous proliferation [2]. Therefore, glycolysis fulfills the most basic requirements of tumors.

Carcinogenesis and cancer progression modulated by tumor glycolysis are arousing considerable interest. However, the immune regulatory role of glycolysis remains unclear. The high concentration of lactate produced by the glycolytic process in TME suppresses anticancer immune cells by disturbing their intracellular pH [32,33]. Furthermore, cancer glycolytic activity competes with immune cells for glucose uptake [34,35], whereby cancer cells effectively achieve immune evasion. We found that increased tumor glycolytic activity is related to poor tumor lymphocyte infiltration in most of the studied cancer types. Previous studies also state that it is inversely associated with the tumor infiltration of T cells in melanoma, non-small-cell lung carcinoma (NSCLC), and HNSC samples [15,36]. As for the TH cells in the current work, increased glycolysis scores strongly correlated with high TH2 and low TH1 cell infiltration, which is an interesting phenomenon. Preferential accumulation of immunosuppressive and TH2 cells, rather than antitumor TH1 cells, are vital for tumor immune evasion [37,38]. Similarly, the relationships between glycolytic activity and the PD-L immune checkpoint and TCR richness also suggest that increased glycolysis promotes tumor proliferation by immune evasion.

We queried Cmap using the gene expression signatures from the glycolysis-associated gene analysis. Surprisingly, the Cmap analysis, which is based on a limited number of treated cell lines, very precisely selected drugs that have been shown to affect cancer metabolism with specificity. MTOR inhibitors were significantly enriched in all cancer types and have been reported to inhibit glycolysis-related tumorigenicity [39]. Interestingly, mTOR actively participates in T cell function and activity [40]. Dopamine receptor antagonists, the most frequent compounds in the Cmap analysis, also showed a capacity to inhibit tumor progression [41]. These translational analyses may provide alternative approaches to metabolic cancer treatment.

Different cancers have similarities in cell cycle, energy substance synthesis, cell proliferation, and cancer progression. Pan-cancer research aims to study these common pathways from the perspective of molecular microbiology to develop a cancer treatment method that has the same treatment for different diseases. Altered metabolism is a hallmark of cancer, and glycolysis is one of the important factors promoting tumor development. In the present study, high-glycolysis-score tumors were associated with worse prognoses across cancer types. Tricarboxylic acid (TCA) cycle, DNA replication, tumor proliferation, and other cancer hallmarks were more active in glycolysis-high tumors. Growth signals, oncogene mutation, and other potential signals could activate glycolysis, thereby regulating the cell cycle, energy material synthesis, cell proliferation, and cancer progression.

Our study provides a comprehensive view of glycolysis-associated molecular signatures, including genome, transcriptome, and proteome data. Furthermore, we quantified 8633 patients across different cancer types from TCGA and evaluated their prognostic associations. The prognostic significance of glycolysis was then validated using three other independent datasets.

## Conclusions

In summary, glycolysis could be a reliable predictor of the clinical outcomes of tumors. This study also provides a comprehensive catalog of molecular alterations associated with glycolysis and contributes to our understanding of glycolysis, thereby potentially leading to effective survival prediction, treatment decision-making, and target therapy identification.

## Data Availability

The datasets analyzed in this study were obtained from the TCGA database (http://www.cancer.gov/tcga).
